# Research Progress on Cell Membrane-Coated Biomimetic Delivery Systems

**DOI:** 10.3389/fbioe.2021.772522

**Published:** 2021-11-16

**Authors:** Mengyu Guo, Chenjie Xia, Yu Wu, Nong Zhou, Zhipeng Chen, Weidong Li

**Affiliations:** ^1^ College of Pharmacy, Nanjing University of Chinese Medicine, Nanjing, China; ^2^ Engineering Center of State Ministry of Education for Standardization of Chinese Medicine Processing, Nanjing University of Chinese Medicine, Nanjing, China; ^3^ The Chongqing Engineering Laboratory for Green Cultivation and Deep Processing of Three Gorges Reservoir Area’s Medicinal Herbs, College of Food and Biology Engineering, Chongqing Three Gorges University, Chongqing, China

**Keywords:** cell membrane, biomimetic, drug delivery, nanomedicine, nanobiotechnology

## Abstract

Cell membrane-coated biomimetic nanoplatforms have many inherent properties, such as bio-interfacing abilities, self-identification, and signal transduction, which enable the biomimetic delivery system to escape immune clearance and opsonization. This can also maximize the drug delivery efficiency of synthetic nanoparticles (NPs) and functional cell membranes. As a new type of delivery system, cell membrane-coated biomimetic delivery systems have broadened the prospects for biomedical applications. In this review, we summarize research progress on cell membrane biomimetic technology from three aspects, including sources of membrane, modifications, and applications, then analyze their limitations and propose future research directions.

## Introduction

Traditional drug delivery primarily involves oral, intravascular, muscular, inhalational, or subcutaneous routes. In these drug delivery methods, the delivered drugs pass through the circulation, following which most are eliminated by the kidney resulting in undesirable pharmacokinetics. However, nanoparticles (NPs) can be utilized to overcome this issue. Synthetic NPs have the advantages of increased drug loading, special release properties, and diverse engineering functions; therefore, they are widely studied and used in biomedicine (Swain et al., 2016). However, there remain limitations such as immunogenicity in applying NPs (Blackman et al., 2021). NPs are initially coated with a variety of polymer molecules, including natural substances such as polysaccharides or semi-synthetic substances such as polymers, which are used to make the NPs as biofriendly and immune system evasive as possible. However, the use of polymer invisibility cloaks, such as polyethylene glycol, cannot completely avoid immune clearance, and NPs can still activate the human complement system (Tasciotti et al., 2008; Chiappini et al., 2010; van de Ven et al., 2012). This necessitates the design of novel drug carriers which can potentially improve drug loading capabilities, the ability to avoid immune clearance, self-targeting functions, and controllable drug release activity.

Cells are the basic unit of life’s activities and it the normal functioning of individual cells and cellular systems is crucial for a living body. Researchers have sought inspiration from and attempted to imitate natural cells to construct bionic nano-drug delivery carriers. A biomimetic system coated by a cell membrane, was first established by Hu et al. (2011); they coated erythrocyte membranes with prepared polymer NPs to achieve the biocompatibility of NPs. Since then, this area of research has evolved to allow cell membranes to play additional roles as protective carriers. Herein, we review cell membrane sources, membrane modifications, and applications of cell membrane-coated biomimetic systems.

## Membrane Sources

**FIGURE 1:**
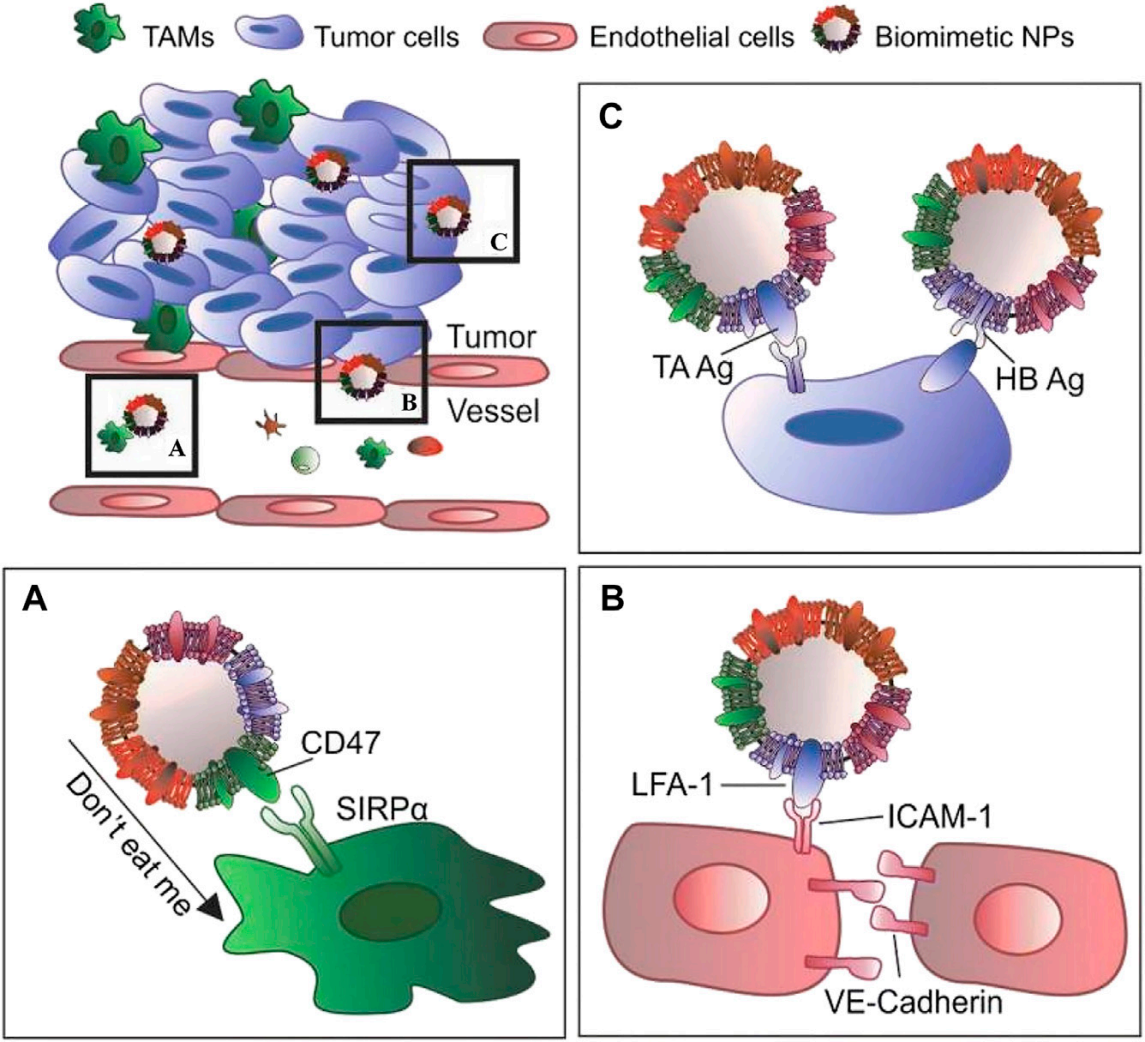
The main functions of the outer cell membrane include: **(A)** protecting drug-carrying NPs from the body’s immune system; **(B)** specific binding with the target cell receptor to achieve of activation or inhibition; **(C)** specifically target tissues and lesion sites to improve efficiency of drug delivery system (Reproduced from Pasto et al., 2019).

The outer cell membrane is not only a simple drug carrier but also a valid shield, and its primary functions include protecting drug-carrying NPs from the body’s immune system, acting as a drug delivery system, and maintaining maximum efficacy. Other functions include targeting, allowing the drug to target specific tissues and lesion sites, and creating a highly efficient drug delivery system (Pasto et al., 2019) (Figure 1). The functions depend on the presence of specific, active membrane proteins.

**FIGURE 2:**
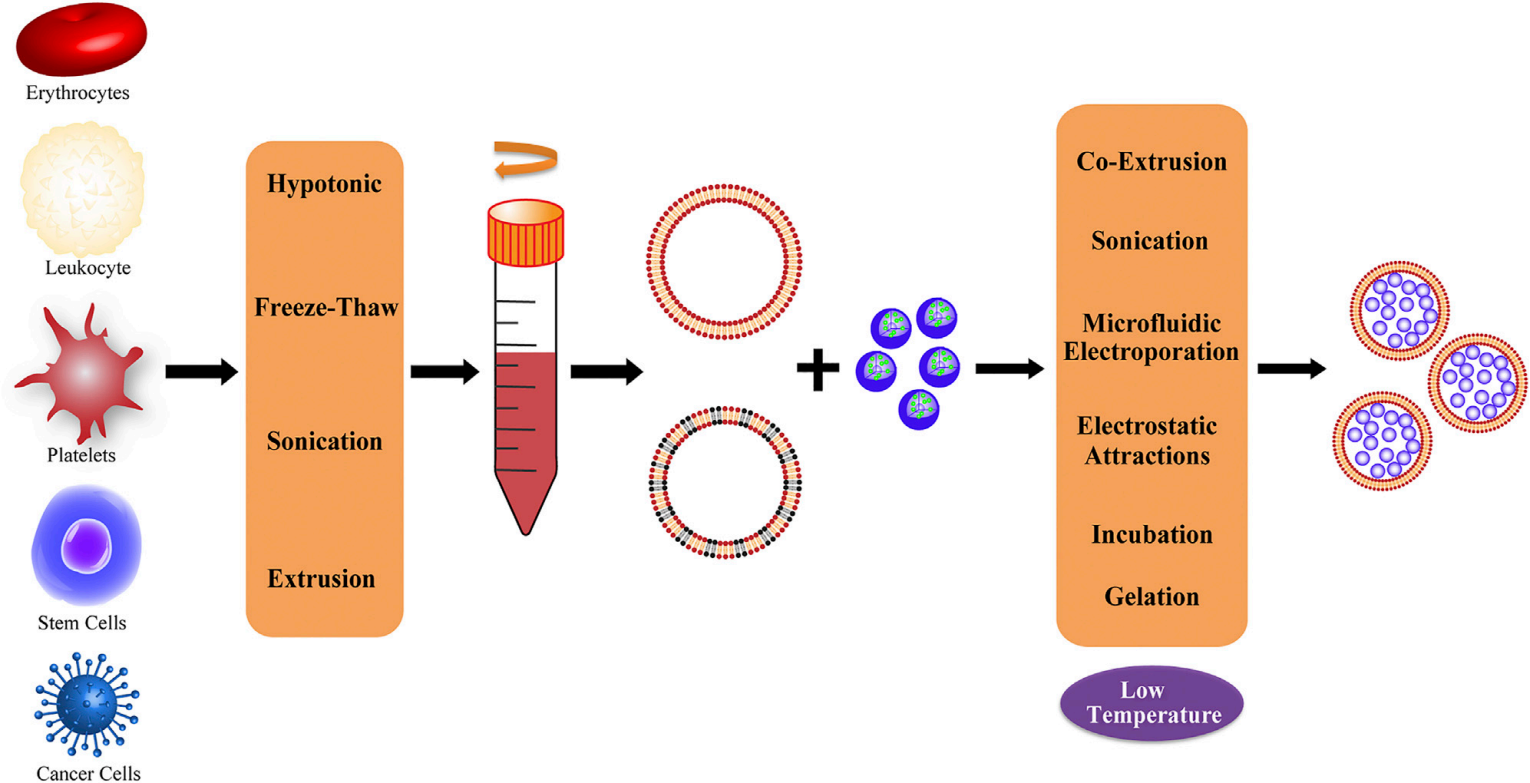
Representative of different cell membrane sources and common extraction methods; the obtained membranes can be processed to produce hybrid membranes.

Current research on cell membranes includes erythrocyte membranes, leukocyte membranes, platelet membranes, stem cell membranes, cancer cell membranes, and hybrid membranes (Liu et al., 2019; Dash et al., 2020) (Figure 2). The complex antigenic characteristics presented on the membrane result in the display of different properties by each type of membrane. Each classification describes different biomimetic properties, and we have summarized the primary features to facilitate convenient comparison (Table 1).

**TABLE 1:** Summary of the differences among different cell membranes.

Source cell	Main properties	Targeting ability	Representative protein
Erythrocytes	Long circulation life	RES-targeting	CD47
	The most abundant blood cell		
	Immune evasion		
Leukocyte	Recognize inflammation	Inflammatory-targeting; Diseased-sites	CD47, CD45, LFA-1, Mac-1
	Circulating tumor cells		
	Immune evasion		
Platelets	The smallest circulating blood cell	Tumor targeting; Injured-sites	P-Selectin, CD41, CD42b, CD61
	Adhesion to other cells		
	Accumulate in diseased region		
	Immune evasion		
Stem cells	Multipotent differentiation	Tumor targeting	Specific integrins
	Self-replication		
	Tumor-specific targeted		
	Immune evasion		
Cancer cells	Unlimited replication	Tumor targeting	Adhesion molecules
	Homologous targeting		
	Immune evasion		

### Erythrocytes

Red blood cells (RBCs) are natural cells with a long circulation time through the body. Their circulation life is up to 100–130 days (Kuhn et al., 2017), and they are the most abundant of all blood cells. Some of their advantageous features are: good plasticity and elasticity, oxygen carrying and some immune capacity, and the ability to pass through capillaries with smaller diameters by changing their shape (Tan et al., 2013; Wicki et al., 2015). One of the reasons RBCs are considered an ideal membrane modifier is because the surface of the RBC membrane is rich in proteins, glycans, and receptors that can bypass attacks by the immune system (Rao et al., 2017; Ren et al., 2017; Su et al., 2017). For example, one of the most representative markers, CD47, is an immunomodulatory protein that acts on the inhibitory receptor SIRPα to prevent clearance from the bloodstream by macrophages (Oldenborg et al., 2000; Legrand et al., 2011; Xie et al., 2019). The RBC membrane was the first to be used to form cell membrane-coated NPs, which Hu et al. (2011) first obtained and successfully prepared utilizing the “invisibility” function and circulation properties of the RBC membrane in 2011. Wang et al. (2019) achieved the targeted release of encapsulated drug-loaded NPs by utilizing the biomimetic properties of the RBC membrane interface to reduce phagocytosis by macrophages and enhance the accumulation of NPs in atherosclerotic plaques.

### Leukocyte

Unlike RBCs, which cannot perform selective targeting, white blood cells (WBCs) are activated by chemokines that are overexpressed in inflammatory tissue, and can recognize inflammation and purposefully accumulate in the lesion area, representing a pivotal marker of the response to disease (Gao et al., 2016b). WBCs, including granulocytes, monocytes, and lymphocytes, can also migrate between extravascular tissues and vessels. Moreover, they have been reported to have targeting abilities against circulating tumor cells through inherent cell adhesion molecules on their membranes (Corbo et al., 2015). Similar to other cells, WBCs are also rich in active functional proteins, such as CD47 and CD45, which confer immune tolerance (Molinaro et al., 2016; Martinez et al., 2018), and lymphocyte function-associated antigen 1 (LFA-1) and macrophage-1 (Mac-1), which allow binding to inflamed endothelium and tumor targeting (Parodi et al., 2013; Palomba et al., 2016). Targeted NPs were prepared for esophageal cancer, including tests using doxorubicin (DOX) and small interfering RNA that interfered with the overexpression of *the LPCAT1* gene. To improve targeting and anti-tumor effects, a white cell membrane coating was used to demonstrate that the coated NPs had more significant anti-tumor proliferation, migration, and metastasis, which was mainly attributed to LFA-1 expressed by WBCs. LFA-1 significantly promotes adhesion between cells and promotes the tumor penetration and internalization ability of the bionic system (Jun et al., 2020).

### Platelets

Platelets are the smallest circulating blood cells and cytoplasmic fragments produced by mature megakaryocytes in the bone marrow (Fang et al., 2014); they play a crucial role in vascular injury, wound healing, inflammatory reactions, and hemostasis after thrombosis (Harker et al., 2000). Platelets have complex properties; for example, there are many proteins on the surface of platelets that can adhere to other cells. Therefore, platelets are closely related to cancer, cardiovascular disease, infection, and other diseases. Especially in cancer, platelets can be recalled and accumulate due to inflammation in the tumor. CD40L, an inflammatory enhancer protein, plays an important role in T cell immunity and dendritic cell maturation (Hu et al., 2017). P-selectin, mainly found in endothelial cells and platelets, is a cell adhesion protein that interacts with cancer cells. It can be exposed on the surface of platelet membranes and specifically binds to the CD44 receptor on the surface of cancer cells (Bergstrand et al., 2019). Additionally, CD41, CD42b, CD61, and platelet membrane glycans are important active components in the interaction between platelets and tumor cells (Li et al., 2016). Activation of these representative proteins mediates NP accumulation at the tumor site, which reduces vascular inflammation and increases drug accumulation and therapeutic effects. Inspired by these properties of platelets, Hu et al. (2015) developed a functional NP-coated platelet membrane to enhance anti-tumor efficacy, which has targeting and site-specific abilities allowing the delivery of extracellular drugs and intracellular small molecules. Moreover, the biomimetic delivery system can effectively clear circulating tumor cells from the body and inhibit the occurrence of tumor metastasis (Hu et al., 2015).

### Stem Cells

Stem cells are multi-potential differentiated cells that have a strong ability to self-replicate. Many types of stem cells, such as bone marrow mesenchymal stem cells and neural stem cells, have been proven to have the ability to target tumors and are widely used in tumor-specific targeted transport. Mesenchymal stem cells (MSCs) exist in diverse tissues and retain their original biological properties after extraction, even after continuous subculture and cryopreservation (Timaner et al., 2018). In addition, MSCs possess an effective homing ability which is believed to be the cause of chemotaxis to the site of injury or stimulation. The tumor-targeting of MSCs is associated with surface-specific integrins (Toledano Furman et al., 2013). In addition, the NP bionic system prepared using MSC membranes can effectively avoid clearance by the immune system, enhance the tumor-targeting and anti-tumor chemotherapy efficacy of loaded doxorubicin (DOX), and also exhibits long-term stability (Gao et al., 2016a).

### Cancer Cells

Compared with blood cells, cancer cells have unique unlimited replication potential and homologous targeting ability. Because of the proliferation ability of cancer cells, it is easy to obtain cancer cells through cell culture *in vitro*, rather than from autologous plasma or donors (Hanahan and Weinberg, 2011). Biomimetic membrane carriers can camouflage nanodrugs as cancer cells, and use the characteristics of mutual recognition and adhesion of molecules on the surface of cancer cells to actively target drugs to the lesions, thereby achieving drug enrichment and effective treatment. Zhu et al. (2016) encapsulated NPs with specific cell membranes of multiple tumor cell lines, and *in vitro* experiments indicated good self-recognition internalization and immune evasion of the source tumor cell lines. More importantly, the same tumor cell line enables highly tumor-selective self-targeting of homologous tumors *in vivo*, even in the context of a heterotypic tumor (Zhu et al., 2016).

### Hybrid Cells

With the deepening of research and the expansion of applications, certain limitations associated with the use of a single membrane have been identified; thus, researchers have been working to develop two types of membrane fusion coatings, thereby integrating additional advantages and enabling NPs to inherit and amplify the characteristics of both source cells (Zhu et al., 2016; Liang et al., 2018). Dehaini et al. (2017) first adopted the fusion cell membrane strategy, proving that a mixed membrane using RBC and platelet membrane-coated PLGA NPs possesses the dual functions of prolonging blood circulation time and targeting tumors. The mixed cell membrane-modified drug-loaded nanosystem further improved the functionalization of the original drug-loaded nanosystem. Bu et al. (2019) developed Fe_3_O_4_ NPs encapsulated by tumor stem cell and platelet fusion membranes which have strong immune evasion, tumor-targeting activity, magnetic resonance imaging and photothermal therapy function, and can be used to enhance photothermal therapy for head and neck squamous cell carcinoma. Therefore, it is reasonable to conjecture that the hybrid membrane is powerful and has the potential to produce a new nanosystem of multi-membrane coatings with the potential to surpass their single-membrane counterparts.

## Modification of Cell Membrane

**FIGURE 3:**
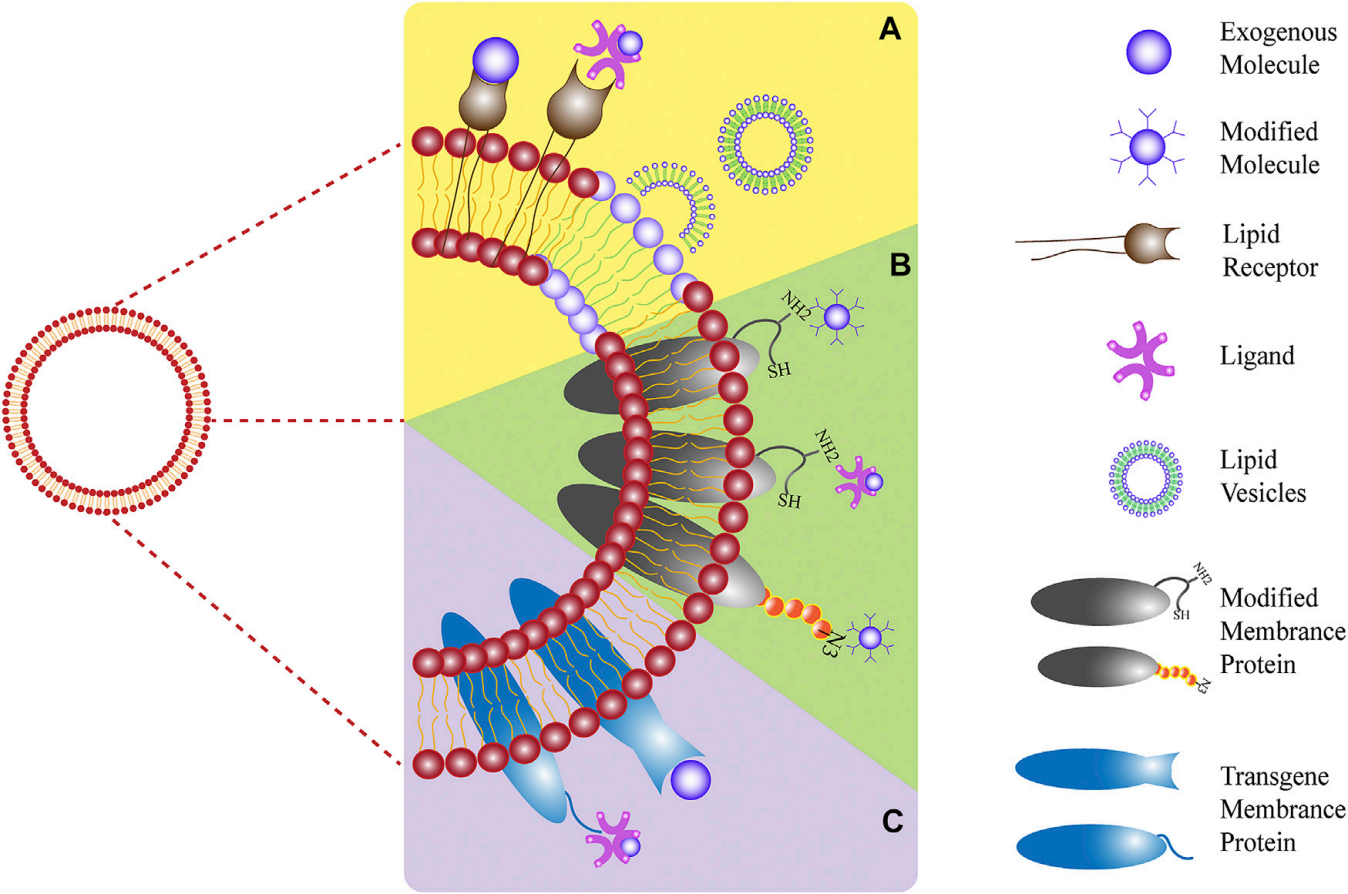
Three modification strategies: **(A)** physical, **(B)** chemical, and **(C)** biological methods.

The cell membrane is composed of lipids, polysaccharides, and proteins, and the functions entrusted to NPs mostly depend on their surface functional proteins. With the latest developments in materials and medical science, the targeting and monitoring operations of nanoplatforms based on cell membranes are increasing. To improve the characteristics of membrane-coated NPs compared to those of natural cell membranes and conduct more precise targeted research and treatment, the modification of cell membranes is a new research direction with substantial potential (Mayor and Etienne-Manneville, 2016; Lim and June, 2017; Yan et al., 2019). Generally, modifications to cell membranes can be roughly divided into three main sections: physical, chemical, and genetic engineering (Bachir et al., 2017) (summarized in Figure 3).

### Physical Engineering of Cell Membranes

**FIGURE 4:**
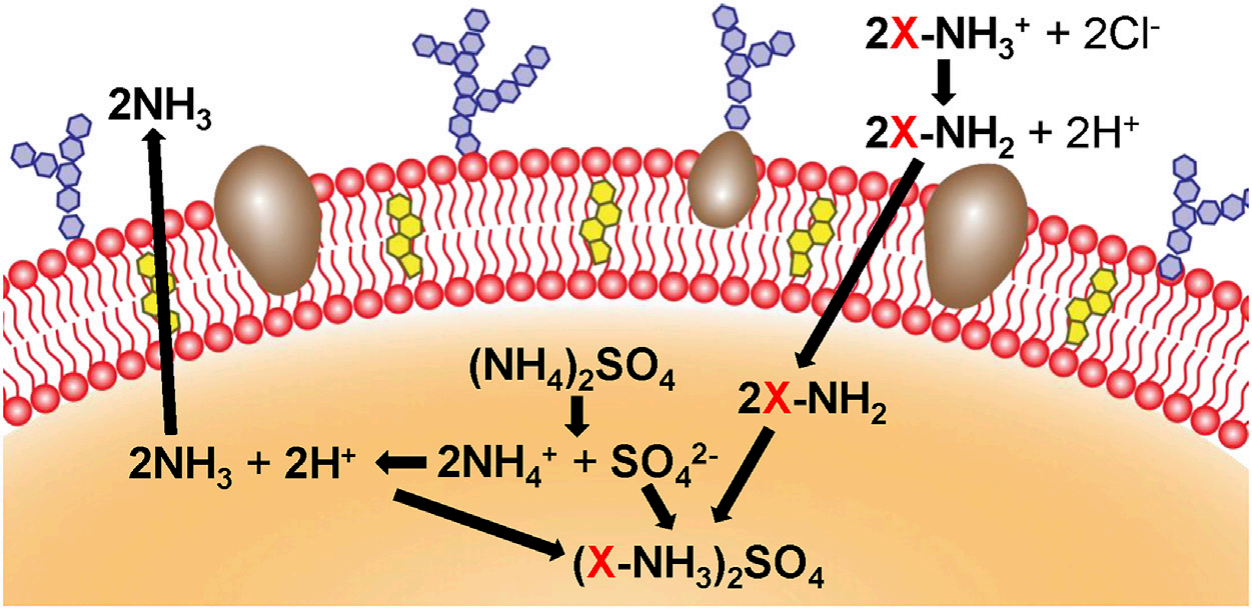
Cholesterol (yellow) and ammonium sulfate (orange) are used to generate a pH gradient, which contributes to the accumulation of the drug in cholesterol-rich RBC vesicles (Reproduced from Zhang et al., 2017).

The physical engineering of cell membranes involves the utilization of lipid structure and membrane fluidity to naturally anchor targeted groups to the cell membrane through lipid-lipid interactions (Escribá et al., 2015). A design for targeted or therapeutic cell membrane can be achieved by inserting the hydrophobic lipid portion, with targeting or therapeutic molecules, into the outer lobule of the lipid bilayer. In addition, exogenous receptors can be inserted into the cell membrane to specifically bind to representative molecules, such as the ligands of probes, therapeutic drugs, and biological macromolecules. Moreover, the fusion of lipid vesicles containing target molecules with cell membranes is a feasible method. For example, glycosylphosphatidylinositol-fused proteins can be designed to attach to the cell membrane, and liposomes can be introduced to fuse with vesicles derived from cell membranes to promote the encapsulation and controlled release of small molecules (Escribá et al., 2015; Saha et al., 2016). It has been reported that vesicles derived from RBC membranes were first fused with cholesterol to encapsulate the chemotherapeutic drug DOX and the antibiotic vancomycin, which exhibited a pH-dependent drug release behavior. And the Exogenous cholesterol supplementation can effectively maintain the pH gradient and drive drug loading (Zhang et al., 2017) (Figure 4). There have also been studies using the method of lipid insertion to achieve recombinant proteins that improve the targeting ability of the membrane bionic system (Zhang Z. et al., 2018). Overall, the physical engineering strategy is convenient and compatible with other membrane modification schemes. However, potential instability of the inserted molecule and a negative effect of the inserted molecule on the overall stability, may limit the effectiveness of this strategy.

### Chemical Engineering of Cell Membranes

**FIGURE 5:**
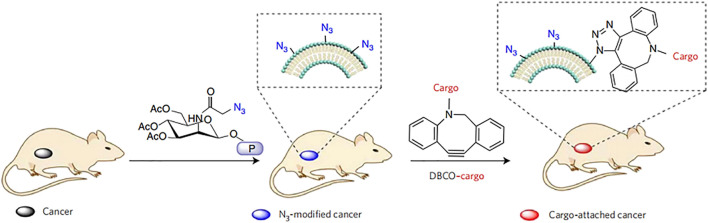
Active tissue targeting via anchored click chemistry technology: *in vivo* selective labeling of cancer cells with azido groups and subsequent cancer targeting via efficient click chemistry (Reproduced from Wang et al., 2017).

Chemical engineering strategies can be used to conveniently endow cells with new functions while preserving their biological competence. Chemical engineering strategies mainly target the primary amine and thiol residues of membrane-associated proteins and the hydroxyl residues of polysaccharides, which can be used as active sites for various covalent conjugation schemes (Sletten and Bertozzi, 2009). For example, through amidation reactions, functional molecules containing carboxyl groups can be combined with the amino residues of cell membranes. It has been reported that tetraacetyl-N-azidoacetylmannosamine-mediated click chemoselective labeling of target cells with azide groups is followed by click chemistry to enhance the accumulation of conjugates (Wang et al., 2017) (Figure 5). Moreover, the strategy of modifying RBC membranes with human recombinant hyaluronidase enabled human recombinant hyaluronidase PH20 to be stably fixed to the extracellular domain of erythrocyte membrane proteins via the cell impermeable linker NHS-PEG-maleimide. Because hyaluronidase overexpression has been found in cancer studies and can reduce the effective drug concentration of treatment, anchoring hyaluronidase to NP-coated cell membranes could potentially improve the spread of NPs in tumors and increase the access of biomimetic systems to tumor cells (Zhou et al., 2016). Thus, chemical strategies are generally convenient and can provide cells with new functions while maintaining their biological functions. However, a limitation is the lack of specificity of the reaction, which may impair the biological activity of the natural protein and affect the original function.

### Biological Engineering of Cell Membranes

**FIGURE 6:**
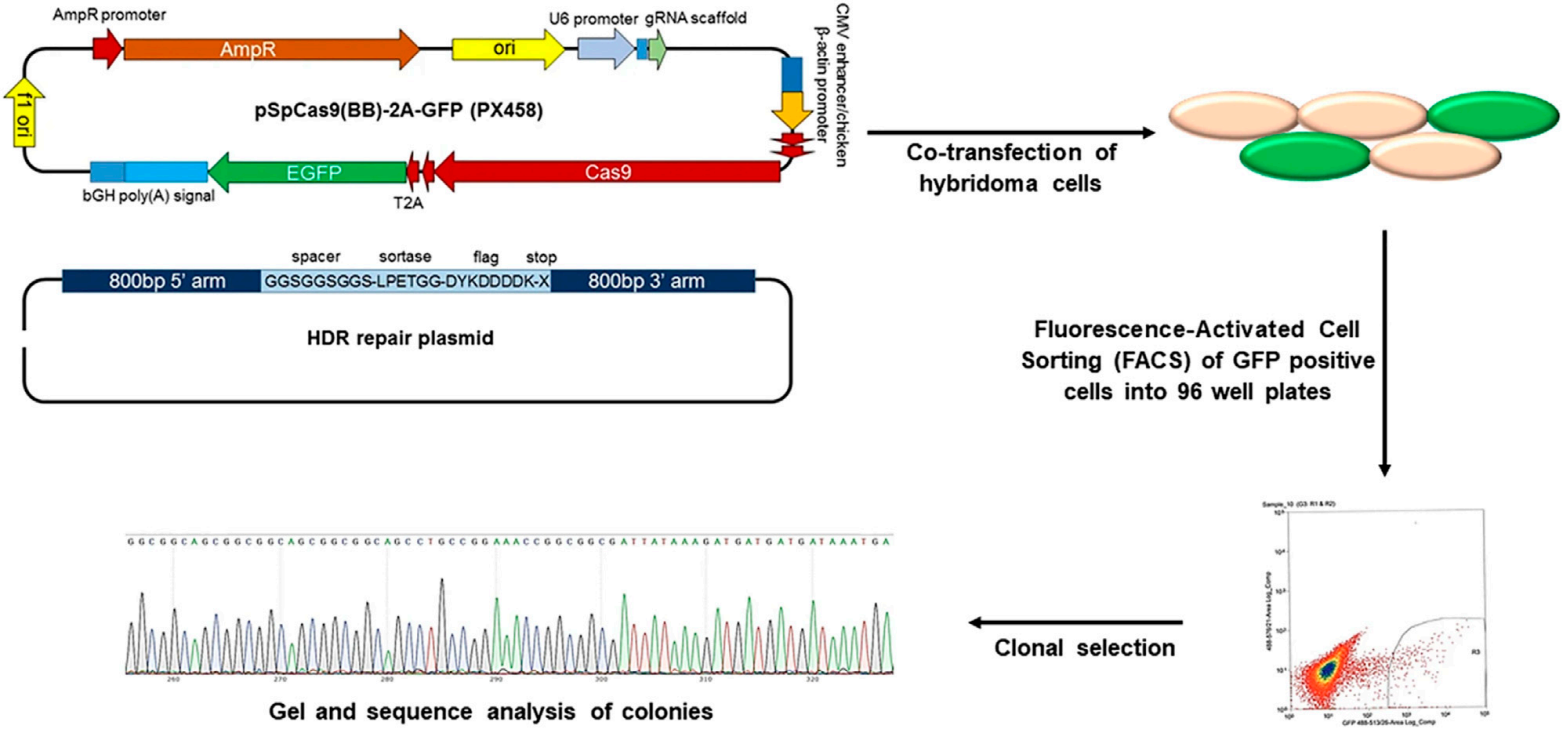
CRISPR/Cas9 genome editing of hybridoma cells for site-specific modification (Reproduced from Khoshnejad et al., 2018).

Genetic engineering is the selective introduction of the desired protein or peptide into the cell membrane through the transfection or transduction of non-viral or viral vectors (Choi et al., 2008; Levy et al., 2013). This strategy is widely used to modify cell membranes with fused motifs for targeting and therapeutic applications (Kell et al., 2015). The thioester acyl residues of the LPETGG motif cleaved by sortase A can connect the N-terminal glycine-functionalized peptide to the cell membrane (Chen et al., 2005). Furthermore, the clustered regularly spaced short palindromic repeats (CRISPR)/CRISPR-associated protein 9 (Cas9) system can be used to promote the introduction of receptor peptides, which allows targeted genome editing with specific guide RNA. The LPXTG motif is genetically expressed on the RBC membrane through the CRISPR/Cas9 system, and the immunodominant peptide containing appropriately exposed N-terminal glycine can be covalently linked with the LPXTG motif with the help of sortase A. This system demonstrated therapeutically efficacy in autoimmune encephalomyelitis (Figure 6) (Khoshnejad et al., 2018; Lao et al., 2018). In general, genetically engineered cell membranes have higher specificity, which could result in more precise cell membrane engineering by combining other chemical or physical strategies. However, both viral and non-viral methods may cause uncontrolled toxicity or immunogenicity.

## Application of Cell Membrane Biomimetic Systems

### Cancer Treatment

The treatment of cancer has always been a major focus and difficulty in scientific research, and the generation of new scientific technologies can be applied to basic research. Previously, although the drug delivery of NPs improved drug loading and control the release of drugs, the targeting and immune clearance of foreign substances prevented the NPs from being widely implemented in clinical applications, despite promising results in a large number of basic studies (Narain et al., 2017; Yaman et al., 2020). To some extent, the cell membrane biomimetic system technology can improve the deficiencies exhibited by simple NPs. The homologous binding of cancer cells leads to the growth of a tumor mass when cancer cells adhere to each other. Therefore, for the delivery of anticancer drugs, membrane coatings with homomorphic binding mechanisms can target cancer cells (Yaman et al., 2020).

As a shield for NPs carrying drugs, the most practical function of the cell membrane is to reduce the clearance rate *in vivo* and homologous targeting of cancer cell membranes. Bahmani et al. (2021) used a platelet membrane to wrap R848 (toll-like receptor agonists) and fuse the platelet membrane with NPs through ultrasound. An *in vitro* study demonstrated that the system can greatly increase the activity of R848 because of the biomimetic stealth effect produced by the platelet membrane and that coated R848 is more likely to be absorbed by cancer cells and has a longer drug retention time (Bahmani et al., 2021).

Another branch of tumor therapy is photothermal therapy (PTT), which uses the targeting property of photosensitizers after entering the body to absorb infrared light from external interference and convert light energy into heat energy to generate heat in the body. This method is simple and non-invasive and is considered as an adjuvant therapy to enhance chemotherapy and radiotherapy (Yang et al., 2017). A system using both platelet-coated DOX and the photothermal agent IR780 was designed. *In vivo* experiments used near-infrared fluorescence imaging to detect NPs *in vivo* and assess tumor targeting and biological distribution, and showed that the system has a longer retention time and accumulation effect. Further evaluation of the combined thermal therapy and tumor-targeting system found that the tumor growth in the laser NP group was completely restrained compared with the system without thermal ablation (Pei et al., 2020).

The single-membrane approach can no longer meet the needs of research, and the emergence of hybrid membranes combined with various cell membrane functions has excellent prospects. Sun et al. (2020) used the homologous specificity of cancer cell membranes combined with the long cycle time of erythrocyte membranes to prepare mixed membranes coated with DOX-loaded gold nanocages, forming a biomimetic system constructed using a hybrid membrane. This hybrid membrane biological system has a higher stability in maintaining the stable encapsulation of drugs before radiation. The release of DOX was less than 20% within 24 h, and the release of drugs was significantly increased to more than 80% when receiving external near-infrared radiation. Moreover, hybrid membrane-coated NPs exhibit better cellular internalization behavior (Sun et al., 2020).

**FIGURE 7:**
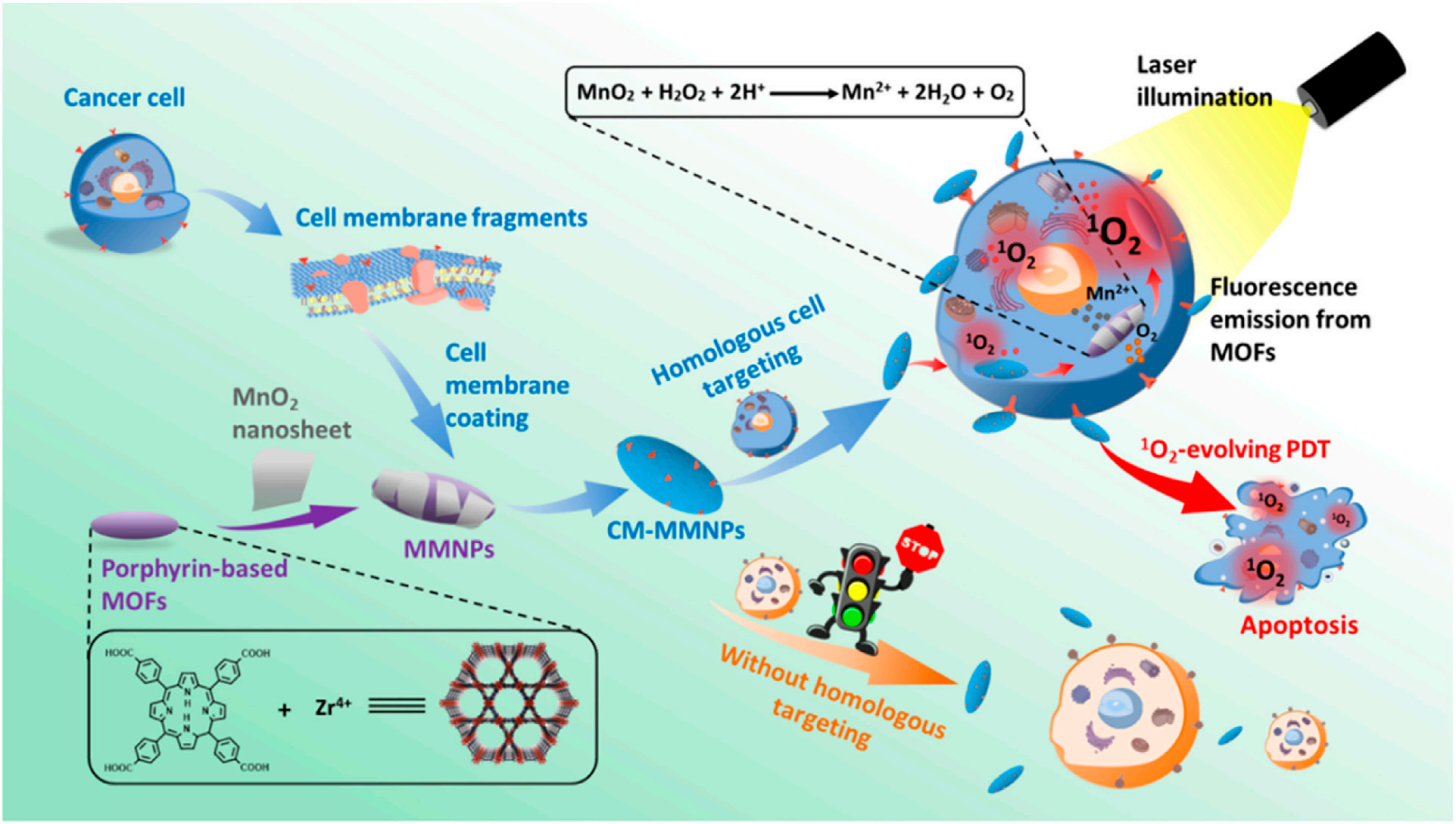
Construction of a biomimetic system with homologous targeting, MRI/fluorescence dual-mode imaging, and PDT (Reproduced from Zhang et al., 2019).

Photodynamic therapy (PDT) is a new choice for cancer treatment. Its anti-tumor effect depends on reactive oxygen species and singlet oxygen produced in the photodynamic reaction, which can producing oxidative reactions and cytotoxicity to kill cells. This process consumes oxygen, so it needs to overcome a certain hypoxic microenvironment. Perfluorocarbons are ideal carriers of oxygen delivery to enhance PDT, and exhibit increased stability when combined with the photosensitizer indocyanine green (ICG) containing human serum albumin. The cancer cell membrane is used for targeting and immune escape, and a biomimetic system has been prepared for scanning imaging and photodynamic therapy. The experimental results showed that the system had a higher oxygen capacity, and fluorescence imaging *in vivo* showed that the fluorescence intensity of the system was stronger and exhibited increased duration, and that the system could significantly inhibit tumor growth (Fang et al., 2021). Zhang et al. (2019) developed novel O_2_-evolved PDT NPs for homologous cancer cell targeting and dual-mode imaging (magnetic resonance imaging and fluorescence imaging). The system comprises a metal-organic framework nucleus coated with a manganese dioxide nanosheet and a cancer cell membrane shell. MnO_2_ layers expressed both H^+^ and H_2_O, and the resulting Mn^2+^ can also be used as an optimal MR contrast agent. The introduction of membranes results in improved stability and integrity during endocytosis, as well as strong targeting of homologous cells (Zhang et al., 2019) (Figure 7).

### Detoxification

**FIGURE 8:**
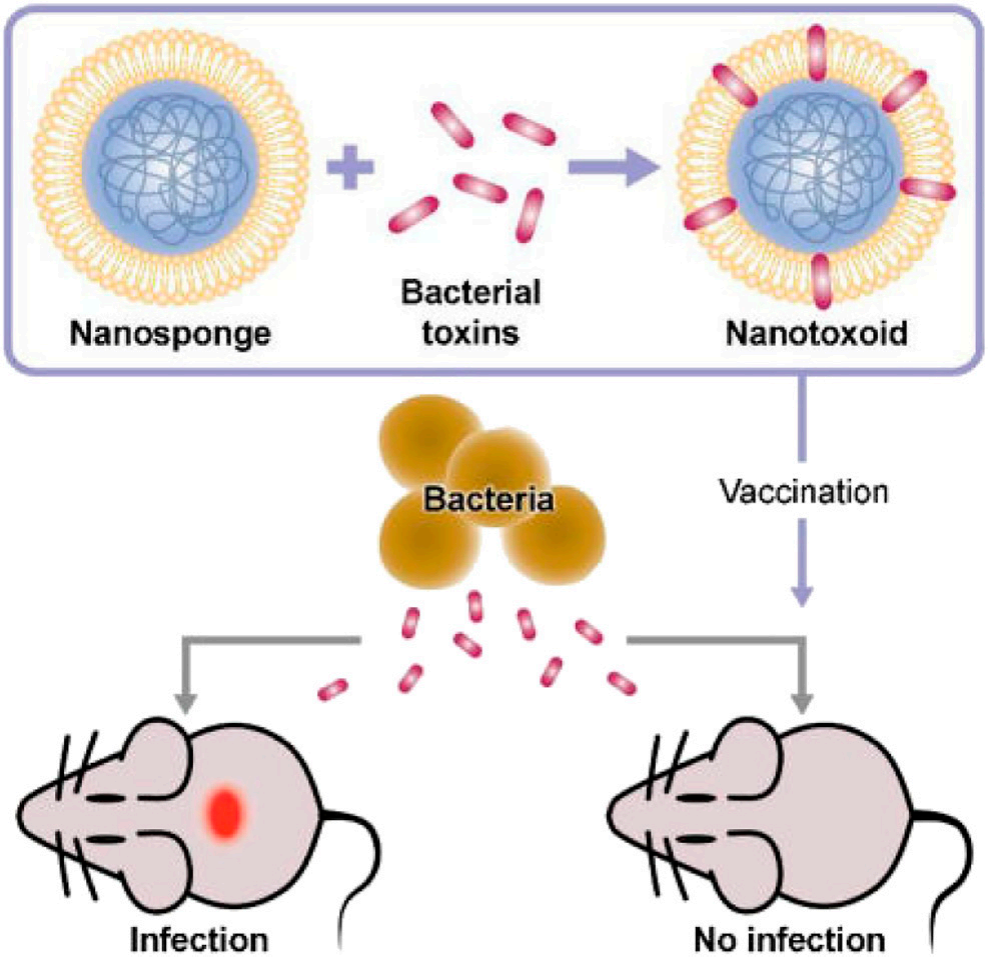
Concept of toxin-NPs (Reproduced from Angsantikul et al., 2018).

Membrane-coated NPs offer a novel strategy to intercept and neutralize bacterial toxins by exploiting the natural affinity of bacterial toxins to the cell membrane. This toxin-NP assembly, known as nanotoxoid, is capable of rapidly loading different types of toxins and has been developed to effectively prevent bacterial infections (Angsantikul et al., 2018) (Figure 8).

Pore-forming toxins are virulence factors secreted by bacteria that can cause considerable damage to host cells by damaging target cell membranes. Therefore, neutralizing or inhibiting the expression of poretoxins is the primary method of improving their antibacterial ability (Kong et al., 2016; Dickey et al., 2017). RBCs have “adsorption” effects on these toxins, and NPs coated with RBCs inherit this function, and thus can be used as a valuable treatment. These particles are called “nano-sponges” and can act as decoys, retaining the toxin inserted into the RBCs and preventing them from lysing cells. This bioactivated toxin nano-sponge offers a detoxification treatment that could potentially treat a variety of injuries and diseases caused by pore-forming toxins. For example, nano-sponges can significantly reduce the toxicity of staphylococcal α-hemolysin and improve the survival rate of mice (Hu et al., 2013). NPs coated by erythrocyte membranes absorb and neutralize bacterial factors associated with bacterial infection; for example, a broad spectrum of programmed toxins can be nonspecifically absorbed and neutralized. Chen et al. (2019) developed and characterized NP-coated RBCs against whole secreted proteins from methicillin-resistant *Staphylococcus aureus* to explore the therapeutic effect of biomimetic systems, and *in vivo* and *in vitro* experiments were conducted assaying hemolytic activity to evaluate the therapeutic effect in WSP-induced lethal phenomena. In addition, they preliminarily explored the mechanism conferring immunity from toxins, and found that it was related to the permeability of the pulmonary endothelium. The molecular mechanism was attributed to the rapid activation of NF-κB (Chen et al., 2019).

In addition, NPs coated on bacterial membranes can stimulate the immune system by simulating the natural bacterial antigens and potentially enhance the immune ability of the body to resist foreign microorganisms (Leleux and Roy, 2013). Gao et al. (2015) developed a natural bacterial membrane collected from bacterial outer membrane vesicles fused onto gold NPs, which showed significantly enhanced stability. When subcutaneously injected, these NPs can rapidly induce the activation and maturation of lymph node dendritic cells. Additionally, immunization with a biomimetic system produced a strong and persistent antibody response with an affinity higher than that induced by membrane vesicles alone (Gao et al., 2015).

### Immune Modulation

Immunity and inflammation are highly correlated; to some extent, inflammation is a form of self-defense, eliminating necrotic cells and tissue. However, in some cases, inflammation can be harmful, such as attacks on healthy organizations (Jin et al., 2018). Autoimmune diseases are caused by an immune response to native components of the body. Under the influence of certain factors, the body’s tissue or the immune system itself can exhibit abnormalities, causing the immune system to mistake its own components as foreign and attack. At this time, the immune system produces antibodies and active lymphocytes aimed at components of the body itself, damaging its own tissues and organs and leading to disease. It is often characterized by the opsonization of target cells by pathological autoantibodies produced by B cells (Eggert et al., 2010).

Cell membrane-coated NPs reserve and present the antigen protein inherited from the cell membrane surface. Therefore, membrane-coated NPs have the ability to act as antibody bait to improve disease parameters, primarily in the context of type II immune hypersensitivity. The pathological immune response is primarily caused by cell lysis or tissue damage, such as in autoimmune hemolytic anemia. Copp et al. (2014) explored the ability of targeted RBC-coated NPs to eliminate viral antibodies, as an alternative target to protect normally circulating RBCs in the body, theoretically eliminating the need for immunodrug therapy and reducing the burden of disease.

**FIGURE 9:**
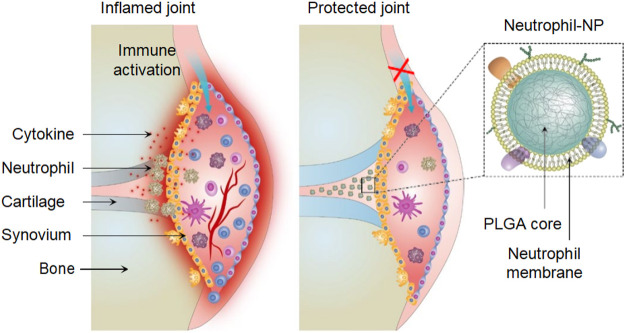
Neutrophil-NPs inhibit synovial inflammation and ameliorate joint destruction in inflammatory arthritis (Reproduced from Zhang Q. et al., 2018).

Rheumatoid arthritis (RA) is a widespread and devastating autoimmune disease. Zhang Q. et al. (2018) designed a neutrophil membrane reprocessing system, which is a human peripheral blood neutrophil membrane coated onto complex NPs that was used for the treatment of RA (Figure 9). In response to cytokine inducers produced in RA, neutrophils accumulate to produce micro-vesicles that enter the cartilage. The combination with NPs cores can neutralize proinflammatory factors, inhibit synovial inflammation, and target the cartilage matrix, resulting in a promising therapeutic effect (Zhang Q. et al., 2018).

Zhang G. et al. (2020) developed NPs encapsulated by CD4^+^ T cells with a polymerist core which were able to target the HIV particle and neutralize not only free HIV but also cell-associated HIV through autophagy. Specifically, CD4^+^ T cells express CCR5 and CXCR4, which are necessary for binding to HIV; thus, the NP-coated T cells were able to selectively bind HIV and also inhibit HIV infection in a dose-dependent manner in human peripheral blood mononuclear cells and mononuclear cell-derived macrophages (Wei et al., 2018; Zhang G. et al., 2020).

### Imaging

The most important roles of cell membrane coating in the imaging field are biocompatibility and homologous targeting of NPs combining photosensitizers and drugs. Li et al. (2020) coated UCNPs with RBCs to ameliorate the limitations of *in vivo* clearance time and immune response, and preserved both the optical properties of UCNP and the immune properties of the cell membrane, thus achieving targeted multimodal imaging. This biomimetic system can be used in magnetic resonance imaging and micro PET/CT imaging of a 4T1 breast tumor mouse model, and showed desirable imaging results and no apparent toxicity *in vivo* (Li et al., 2020).

**FIGURE 10:**
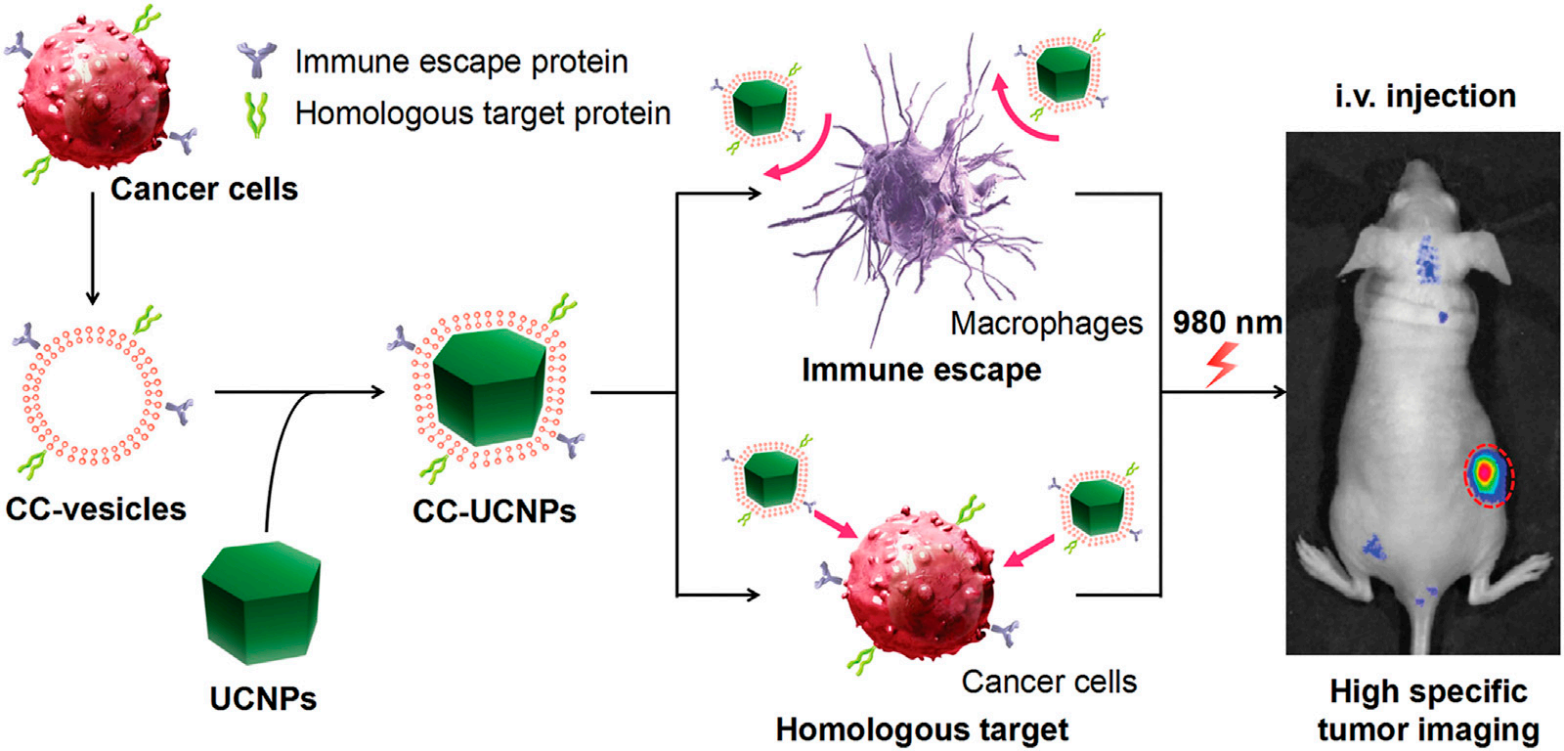
Cancer cell membrane coated UCNPs produce a biomimetic system that exhibit immune escape and homologous targeting capabilities for highly specific tumor imaging (Reproduced from Rao et al., 2016).

Using homologous targeting of cancer cell membranes, the NP-loaded ICG and coated cancer cell membrane, called ICNPS, was prepared by Chen et al. (2016) ICNPS achieved dual-modal imaging using (NIR)-FL/PA. *In vitro* cell experiments showed that ICG had strong fluorescence imaging ability, cell uptake, and targeting and accumulation ability *in vivo*. This bionic system also exhibits potential as photothermal therapy (Chen et al., 2016). Rao et al. (2016) also used cancer cell membranes to wrap UCNPs, protect UCNP from immune clearance, and convert near-infrared radiation into visible light for tumor imaging *in vivo* (Rao et al., 2016) (Figure 10).

Moreover, the ability to target specific locations leads NPs coated with cell membrane systems to be utilized for imaging and PTT. A fibroblast cell membrane with nanomicelles bonded to the biomimetic semiconducting polymer NPs was developed by Li et al. (2018) and called the AF-SPN system. This system can specifically target cancer-associated fibroblasts in the tumor microenvironment and enhance the photothermal characteristics of multimodal cancer. The homologous targeting mechanism enables the bionic system to specifically target tumor-associated fibroblasts, protect SPN to provide better optical signals for tumor detection, and to generate photothermal and photodynamic therapy. The camouflage of cell membranes does not affect the near-infrared fluorescence, photothermal, or photodynamic properties, and the biomimetic system can preferentially target cancer-associated fibroblasts (Li et al., 2018).

However, based on the imaging requirements, the most fundamental improvement method is to modify NPs. To develop more advanced nanoprobes to improve the imaging depth and stability, and to achieve further breakthroughs with the biocompatibility and targeting ability conferred by the cell membrane. For example, Lv et al. (2018) developed a biosystem nanoprobe characterized by the realization of two-photon absorption and Forster resonance energy transfer processes, achieving superior fluorescence performance, including high-resolution visualization of the fine structure of tumor tissue *in vivo*. The cancer cell membrane coating provides biocompatibility with the nanoprobe (Lv et al., 2018).

Therefore, cell-membrane-coated biomimetic delivery systems are widely used. Additionally, the use of Chinese medicine is a good representative of natural medicine, and the excavation and utilization of the active ingredients is a promising breakthrough in Chinese medicine. However, innovative development and utilization of Chinese medicine is often limited by their solubility, stability, and toxicity. Large-sized materials in drug delivery pose some challenges, including *in vivo* stability, bioavailability, solubility, and target-specific delivery problems. Designing cell membrane-coated biomimetic delivery systems by applying cell membrane bionic carriers to deliver active substances may offer a good solution. Research in this field is gradually emerging, and we summarize the application of biomimetic delivery systems to Chinese medicine ingredients combined with cell membranes in Table 2.

**TABLE 2:** Summary of Chinese medicine ingredients used in cell membrane coating research.

Drugs	Membrane	Indications	Strategy	Reference
Curcumin	Cancer cell membrane	Breast cancer	Improve the biosafety and stability of combination photochemotherapy for breast cancer	Zhang et al. (2021)
Curcumin	RBC membrane	Alzheimer’s disease	Load antioxidants into RBC membrane-camouflaged human serum albumin NPs containing T807 and triphenylphosphine, which promote sustained drug release and improve biocompatibility and long-term circulation	Gao et al. (2020)
Curcumin and Angelica polysaccharide	RBC membrane	Hepatocellu-lar Carcinoma	Use cell membrane to avoid immune system clearance	Guo et al. (2021)
Tanshinone IIA	RBC membrane		Prolong the circulation time and prevent oxidative stress at the cellular level by protecting encapsulated drug STS from RES clearance	Dong et al. (2017)
Gambogic acid	RBC membrane	Colorectal cancer	Extend circulation time *in vivo* with improved biocompatibility and stability	Zhang et al. (2020b)
Paclitaxel	Macrophage cell membrane	Melanoma	Improve delivery to cancer metastatic sites	Cao et al. (2020)
Chikusetsusapo-nin IVa	Hybrid cell membrane (RBC membrane and cancer cell membrane)	Breast cancer	Increase immune evasion and targeting ability at the tumor site	Xie et al. (2021)
Resveratrol	RBC membrane	Alzheimer’s disease	Load antioxidants into RBC membrane-camouflaged NPs bearing rabies virus glycoprotein and triphenylphosphine cation, which promote sustained drug release and improve biocompatibility and long-term circulation	Han et al. (2020)

## Conclusion and Perspectives

This review introduces different cell membranes used in cell membrane technology, including erythrocyte membranes, leukocyte membranes, platelet membranes, stem cell membranes, cancer cell membranes, and hybrid membranes. With the widespread application of cell membrane biomimetic technology, modifications of cell membranes are being widely investigated as potential strategies. There are three main types of cell membrane modifications based on the strategy used for modifications: physical, chemical, and biological engineering. The physical engineering of cell membranes mainly uses lipid structure and membrane fluidity to anchor targeted groups to the cell membrane; chemical engineering strategies mainly target active chemical bond reaction sites for various covalent conjugation schemes; and the biological engineering strategies can selectively introduce the desired protein or peptide into the cell membrane through the transfection or transduction of non-viral or viral vectors.

An increasing number of studies are focused on cell membrane encapsulating NPs, which are mainly used for cancer treatment, drug delivery, detoxification, immune regulation, and imaging. Among these, specific targeting is the most important central role in cancer and imaging. In addition, by taking advantage of the inevitable action between toxins and the cell membrane, the cell membrane can be used as a broad-spectrum detoxification agent. Regarding immunity, in addition to the inherent targeting effect of cell membranes, many studies have been carried out in the field of autoimmune diseases using antigen-specific approaches, which are expected to further solve the problem of immunity.

Despite the advantages of cell membrane bionic technology, further improvements are still required. There remains substantial scope for the development and application of engineered cell membrane-based nanotherapeutics. Moreover, there are additional problems that remain to be solved.

First, membrane biomimetic technology is mainly derived from various animals; that is, the membrane is obtained from the animal body, which is dependent on the animal model used in *in vivo* and *in vitro* experimental studies, and the specific clinical efficacy remains to be verified. Studies have used human cells to study human glioblastoma U87 and U87-CXCR4 cell lines at the animal level (Jin et al., 2019). Although the technique does not have extensive clinical applications, its rapid development provides a new direction for the refinement of individual medicine in the future.

Second, the quality control of cell membranes seems to have been ignored. Determining and controlling the critical quality attributes of cell membranes warrants additional research. The characterization of functional proteins on the surface of cell membranes, which is an important source of targeting and homology, is an important indicator in all studies. The source of cell membranes produces differences not only in the different cell types used, but also for in the same types of cells when used from different species. Microscopic characterization is still insufficient to characterize the varying properties of different cell membranes. Many research groups are continuously working to refine and upgrade existing methods and standards to develop industry-wide benchmarks and manufacturing principles. In addition, as for the current cell culture and membrane derivative technology, whether the combination technology of membrane and NPs can cope with high-throughput culture and preparation, and whether the changes in experimental conditions requires the consideration of new standards to measure the purity of membrane derivation remains to be seen.

The membrane modification process is equivalent to killing the cells and resplicing the fragments. Identifying strategies to deal with the body’s removal of cell fragments and better simulate the function of living cells, rather than simple cell membrane extraction and separation, can further improve the function of the membrane bionic system.

Owing to the limited membrane sources, the functional components of the cell membrane can be identified *in vitro* to facilitate clinical transformation. Although there are plans to integrate the active proteins of the cell membrane into the synthetic phospholipid bilayer and use the generated bionic vesicles to achieve targeting (Molinaro et al., 2016), further research in this area is needed.

We hope that by exploring the relationship between critical quality attributes and the function of the carrier and establishing the standardization of cell membrane sources, we can accelerate the clinical transformation of cell membrane-coated NPs, which will have a positive impact on human health and be of great economic value.
